# Atomically precise silver clusterzymes protect mice from radiation damages

**DOI:** 10.1186/s12951-021-01054-5

**Published:** 2021-11-19

**Authors:** Jiao Guo, Haiyu Yang, Ya Liu, Wei Liu, Ruiying Zhao, He Li, Wei Long, Wenqing Xu, Meili Guo, Xiaodong Zhang

**Affiliations:** 1 Tianjin Key Laboratory of Radiation Medicine and Molecular Nuclear Medicine, Institute of Radiation Medicine, Chinese Academy of Medical Sciences and Peking Union Medical College, Tianjin, 300192 China; 2 Department of Physics and Tianjin Key Laboratory of Low Dimensional Materials Physics and Preparing Technology, School of Sciences, Tianjin University, Tianjin, 300350 China; 3 Department of Physics, School of Science, Tianjin Chengjian University, Tianjin, 300384 China

**Keywords:** Ag_14_ clusterzymes, Atomically precise, Renal clearance, Radioprotection

## Abstract

**Background:**

As we know, radiotherapy plays an irreplaceable role in the clinical management on solid tumors. However, due to the non-specific killing effects of ionizing radiation, normal tissues damages would be almost simultaneous inevitably. Therefore, ideal radioprotective agents with high efficiency and low toxicity are always desirable. In this work, atomically precise Ag_14_ clusterzymes were developed, and their applications in radioprotection were studied in vitro and in vivo for the first time.

**Methods:**

The ultra-small glutathione supported Ag_14_ clusterzymes were synthesized by convenient sodium borohydride (NaBH_4_) reduction of thiolate-Ag (I) complexes and then they were purified by desalting columns. The enzyme-like activity and antioxidant capacity of Ag_14_ clusterzymes have been tested by various commercial kits, salicylic acid method and electron spin resonance (ESR). Next, they were incubated with L929 cells to evaluate whether they could increase cell viability after γ-ray irradiation. And then Ag_14_ clusterzymes were intravenously injected into C57 mice before 7 Gy whole-body γ-ray irradiation to evaluate the radioprotection effects in vivo. At last, the in vivo toxicities of Ag_14_ clusterzymes were evaluated through biodistribution test, hematological details, serum biochemical indexes and histological test in female Balb/c mice with intravenous injection of Ag_14_ clusterzymes.

**Results:**

Our studies suggested atomically precise Ag_14_ clusterzymes were potential radioprotectants. Ag_14_ clusterzymes exhibited unique superoxide dismutase (SOD)-like activity, strong anti-oxidative abilities, especially on •OH scavenging. The Ag_14_ clusterzymes could effectively improve cell viability through eliminating ROS and prevent DNA damages in cells dealt with γ-ray irradiation. In vivo experiments showed that Ag_14_ clusterzymes could improve the irradiated mice survival rate by protecting hematological systems and repairing tissue oxidative stress damage generated by γ-ray irradiation. In addition, bio-distribution and toxicological experiments demonstrated that the ultrasmall Ag_14_ clusterzymes could be excreted quickly from the body by renal clearance and negligible toxicological responses were observed in mice up to 30 days.

**Conclusion:**

In summary, atomically precise, ultrasmall and water soluble Ag_14_ clusterzymes with SOD-like activity were successfully developed and proved to be effective both in vitro and in vivo for radioprotection. Furthermore, with atomically precise molecular structure, Ag_14_ clusterzymes, on aspect of the catalytic and optical properties, may be improved by structure optimization on atom-scale level for other applications in disease diagnosis and treatment.

**Graphical Abstract:**

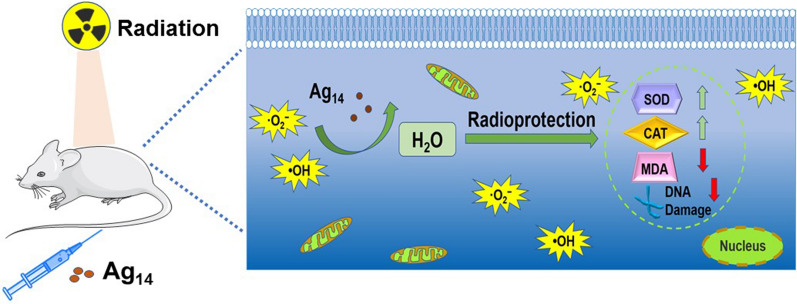

**Supplementary Information:**

The online version contains supplementary material available at 10.1186/s12951-021-01054-5.

## Background

Radiotherapy plays an irreplaceable role in the clinical management on solid tumors [1–6]. However, in the process of radiotherapy, ionizing radiation will inevitably cause some damages to normal tissues, which is mainly attributed to exogenous reactive oxygen species (ROS), such as hydroxyl radical (•OH) and superoxide anion (O_2_^•−^), generated by ionization of water molecules through Compton scattering, photoelectric and Auger effects of high energy ionizing radiation [7–12]. Excessive ROS can react with cellular macromolecules, such as nucleic acids, proteins and lipids, resulting in DNA double-strand breaks (DSBs), proteins and lipids oxidation, and eventually cell death or carcinogenesis [13–18]. Therefore, the development of radiation protective agents with ROS scavenging ability is a feasible solution to reduce radiation damages to normal tissues and improve the therapeutic effects in radiotherapy.

Amifostine and 3, 3-diindolylmethane (DIM) are widely known as radioprotectants for they can scavenge excessive ROS generated by irradiation. However, the blood elimination half-life of these small molecule agents (< 10 min) are too short to afford satisfactory protective effects at a safe dose [19–22]. Thus, it is of great significance to develop new radioprotectants with appropriate long blood elimination half-life. Nanomaterials with relatively large hydrodynamic size provide possibilities to solve this problem. In recent years, nanomaterials with ROS scavenging activity have shown great potential in radiation protection, especially those with rapid renal clearance showing good biocompatibility and low toxicity, such as CeO_2_, WS_2_, and MoS_2_ nanoparticles [23–29]. However, due to their inaccessible molecular structures or heterogeneous size distributions, these nanomaterials face great challenges in improving catalytic activity and bio-selectivity. Meanwhile, replication of these nanomaterials in a large-scale with the same therapeutic effects is still in difficulty [30, 31]. Therefore, it is an urgent task to explore nanomaterials with unambiguous structure–activity relationship for ROS elimination in vivo. Recently, atomically precise noble metal (e.g., Au and Ag) nanoclusters with enzyme-mimic catalytic activities were investigated for catalytic application and treatment of injuries induced by excessive ROS. These nanoclusters were named as metal clusterzymes [32, 33]. For the atomically precise molecular structure and high surface atomic ratio, the clusterzymes have high catalytic activities, and the catalytic activity and selectivity can be regulated at molecular levels. Moreover, the metal clusterzymes are biocompatible, water-soluble and ultra-small scale (generally 1–3 nm) to be excreted through urine, without long-term cumulative toxicity.

Herein, we present atomically precise glutathione (GSH) supported Ag clusterzymes, Ag_14_(GSH)_8_, for the treatment of radiation injuries. To our knowledge, the application of well-defined metal clusterzymes for radiation protection has not been reported yet. As the surface ligand of the Ag_14_ clusterzymes, the glutathione was not only be used to stabilize the ultra-small Ag_14_ core (< 1.5 nm) through its zwitterionic feature, but also afford excellent biocompatibility and anti-oxidation capacity to Ag_14_ clusterzymes [30, 34, 35]. The Ag clusterzymes in this study showed strong antioxidant capacity and ROS scavenging ability, which can prevent cells from viability decrease and DNA damages induced by high-energy γ-ray irradiation. In vivo experiments showed that the Ag_14_ clusterzymes could effectively improve the survival rate, decrease the hematological system and bone marrow DNA damages of irradiated mice. Meanwhile the oxidative stress of major organs was observed to go back to normal levels by Ag_14_ clusterzymes management. The ultra-small glutathione supported Ag clusterzymes could be rapidly excreted out of the body through kidneys and did not cause any toxicological effects in 30 days post injection.

## Results

**Fig. 1:**
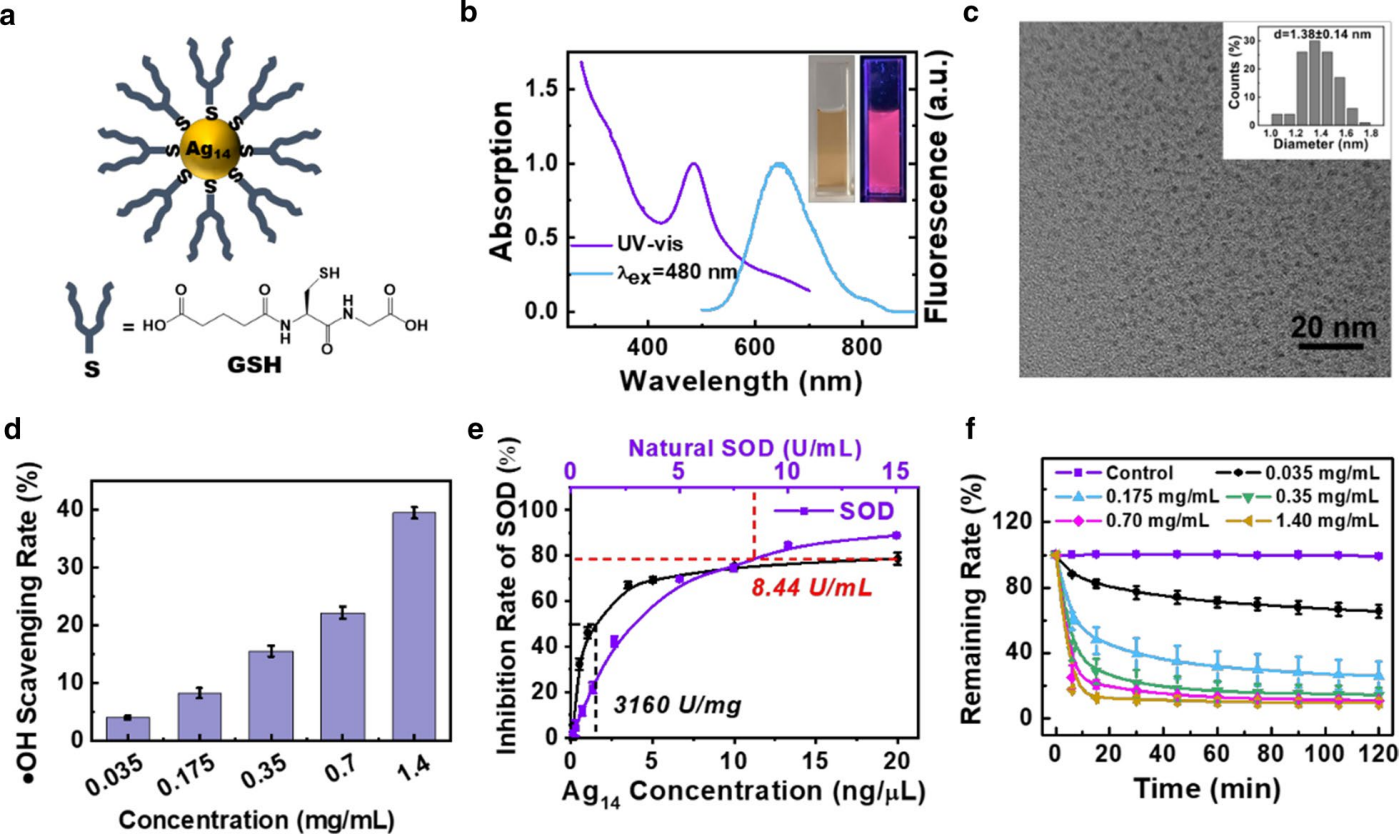
Characterization and antioxidant properties of Ag_14_ clusterzymes. **a** Schematic illustration of Ag_14_ clusterzymes. **b** Optical absorption (purple line) and photoemission (blue line, λex = 480 nm) spectra of Ag_14_ clusterzymes. Insets: photographs of Ag_14_ clusterzymes and the red-emitting Ag_14_ clusterzymes taken under visible and UV light. **c** Typical TEM image of Ag_14_ clusterzymes. Insets: size distribution of Ag_14_ clusterzymes obtained from TEM image. **d** •OH clearance ability of Ag_14_ clusterzymes under different concentrations characterized by SA assays. **e** The SOD-like activity of Ag_14_ clusterzymes at different concentrations characterized by WST-8 and the comparison with natural SOD. **f** Time-dependent total antioxidant capacity of Ag_14_ clusterzymes under different concentrations characterized by ABTS assays

The ultra-small glutathione supported Ag_14_ clusterzymes were synthesized by convenient sodium borohydride (NaBH_4_) reduction of thiolate-Ag(I) complexes and then they were purified by desalting columns according to a literature report [35]. The formation of Ag_14_ was confirmed by absorption and fluorescence spectra in water solution (Fig. 1b). A distinct absorption band peaked at 484 nm was observed (Fig. 1b, purple line), manifesting structure characteristics and size focusing of the as-prepared Ag_14_. The Ag_14_ purified by desalting columns showed obvious red fluorescence peaked at 640 nm (Fig. 1b, blue line), which is in consistent with those reported in literatures [34, 35]. The yield of Ag_14_ was determined to be ~ 80% (based on the amount of Ag in the precursor) by using inductively coupled plasma massspectrometry (ICP-MS). The monodispersity of the product was demonstrated by polyacrylamide gel electrophoresis (PAGE) analysis, which showed only one distinct band (Additional file 1: Figure S1a). Furthermore, the composition of Ag nanoclusters was checked by electrospray ionization (ESI) mass spectrometry. As shown in Additional file 1: Figure S1b, the peak at ~ m/z of 1342 were assigned to [Ag_14_(SG)_8_ + 3Na]^3+^, which is in consistent with those reported in literature [35]. These results indicated that the Ag_14_(SG)_8_ clusters were successfully synthesized. The representative transmission electron microscope (TEM) image of the Ag_14_ in Fig. 1c showed that the Ag_14_ nanoclusters was mono-dispersity in water and have an ultra-small molecular size of ~ 1.4 nm. Due to the strong coordination between Ag atoms and the thiol groups, the Ag_14_ nanoclusters were stable in water and physiological solvent, such as PBS and FBS (Additional file 1: Figure S2).

In recent years, Ag nanomaterials with oxidation properties, such as peroxidase (POD)-like activities and •OH generation abilities, have been employed for antimicrobial applications and cancer treatments [36–39]. However, in this work we found that glutathione supported Ag_14_ clusterzymes exhibited strong antioxidant capacities and ROS scavenging abilities, especially on •OH and O_2_^•−^ scavenging. Salicylic acid (SA) assays were applied to tested the •OH clearance ability of Ag_14_. The salicylic acid solution turns into dark purple rapidly when react with hydroxyl radical. However, the color faded to light purple when Ag_14_ were added, suggesting the hydroxyl radical could be effectively eliminated by Ag_14_ clusterzymes (Additional file 1: Figure S3). As shown in Fig. 1d, the •OH clearance ability of Ag_14_ clusterzymes was concentration-dependent, where 39.4% •OH could be eliminated at a concentration of 1.4 mg/mL (The concentration of the sample shown here and below indicates the concentration of Ag_14_(SG)_8_ clusters, which was calculated according to the content of Ag in a certain volume of solution through ICP-MS).

The O_2_^•−^ scavenging ability of Ag nanoclusters was reported for the first time. The superoxide dismutase SOD-like activity of Ag_14_ clusterzymes was detected by a WST-8 colorimetric method. It was found that the inhibition rate of Ag_14_ clusterzymes on O_2_^•−^ was concentration-dependent, which reached 79.7% at a concentration of 20 ng/μL (Fig. 1e). According to the definition of unit of enzyme activity, the enzyme specific activity of Ag_14_ is high up to 3160 U/mg. We further tested the inhibition rate of 0–15 U/mL natural SOD on O_2_^•−^ and compared with the SOD-like activity of Ag_14_ clusterzymes. As shown in Fig. 1e, the inhibition rate of 20 ng/μL Ag_14_ clusterzymes is comparable to that of 8.44 U/mL natural SOD. The SOD-like activity of Ag_14_ clusterzymes was stable in water, which almost unchanged after 7 days, and only decreased by 14% after 14 days (Additional file 1: Figure S4). We further investigated the •OH and O_2_^•−^ scavenging of the Ag14 clusterzymes by directly assessing •OH and O_2_^•−^ concentrations using electron spin resonance (ESR). As shown in Additional file 1: Figure S5, with increasement of Ag14 clusterzymes concentrations, the ESR signals of the spin adducts, BMPO/•OH and BMPO/•OOH, significantly decrease, which demonstrated increasing scavenging efficiency toward •OH and O_2_^•−^, respectively.

Lastly, the total antioxidant capacity of Ag_14_ clusterzymes was evaluated by 2, 2ʹ-azino-bis (3-ethylbenzthiazoline-6-sulfonic acid (ABTS) assays. Oxidative ABTS^+•^ could be reversibly reduced by antioxidants featured with absorbance decrease at 414 nm. As shown in Fig. 1f, the antioxidant ability of Ag_14_ clusterzymes was time and concentration dependent. And the elimination of Ag_14_ clusterzymes to ROS was rapid, only 17.8% ROS remained after 6 min of incubation at a concentration of 1.4 mg/mL. It is known that the protective ligand glutathione (GSH) is an antioxidant. The antioxidant capacity of Ag_14_ clusterzymes may stem from GSH. In order to study the mechanism of antioxidant activity of Ag_14_, cysteamine (Cys) stabilized Ag nanoclusters were synthesized by the same method. As shown in Additional file 1: Figure S6, the ABTS^+•^ clearance kinetic of Ag_14_ is comparable with GSH at same concentration, but significantly higher than that of the Ag-Cys nanoclusters. Furthermore, GSH stabilized Ag_16_(GSH)_9_ nanoclusters were reported to possess superior antimicrobial properties via generating a high concentration of intracellular ROS [40, 41]. These results indicated that the antioxidant capacity of Ag nanoclusters could not simply attributed to protective ligands with antioxidant capacity, which probably related to the unique molecular structure and coordination interactions between Ag atoms and thiol groups [30, 42–44]. These results demonstrated excellent ROS elimination ability of the Ag_14_ clusterzymes in vitro and it could be used for radiation protection.

**Fig. 2:**
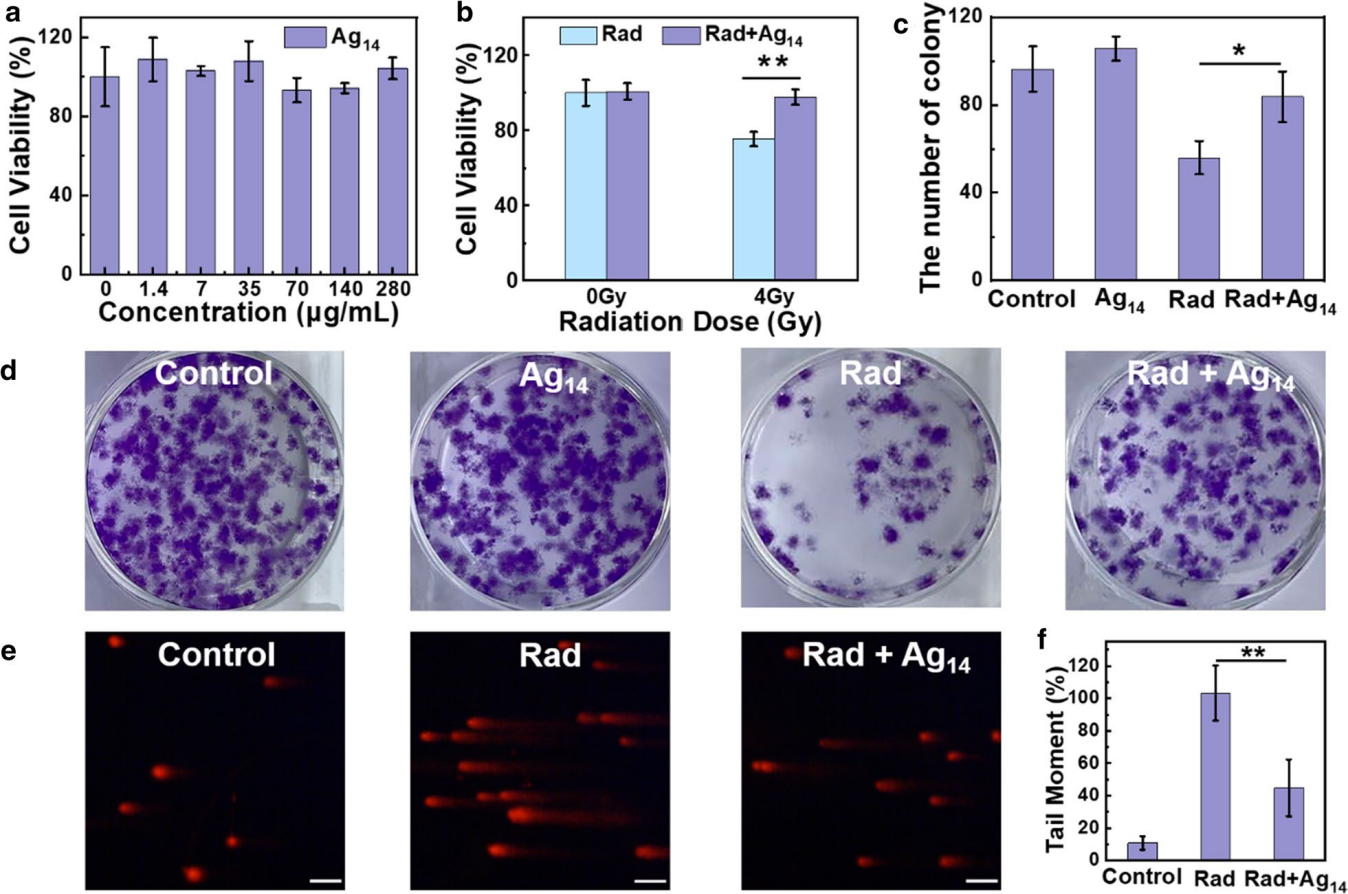
Cytotoxicity and radiation protection of Ag_14_ clusterzymes in vitro. **a** Viability of L929 cells after incubation with Ag_14_ clusterzymes at doses from 0 to 280 μg/mL for 24 h. **b** Viability of L929 cells treated with or without Ag_14_ clusterzymes (280 μg/mL) after radiation at the dose of 0 and 4 Gy gamma ray. **c**, **d** Colony assay of L929 cells treated with or without Ag_14_ clusterzymes (280 μg/mL) upon radiation at a dose of 2 Gy. **e**, **f** Comet images of L929 cells treated with or without Ag_14_ clusterzymes (280 μg/mL) after radiation at a dose of 4 Gy, the tail length positively correlated to DNA damages in cells. Scale bars are all 50 μm. p values: ***p < 0.001, **p < 0.01, or *p < 0.05, ANOVA

The in vitro radiation protection performance of Ag_14_ clusterzymes was then studied by a band of cell experiments. Cellular uptake of Ag_14_ clusterzymes in mouse fibroblast L929 cells after 1 h incubation demonstrated efficient cellular uptake of Ag_14_ clusterzymes (Additional file 1: Figure S7). As shown in Fig. 2a, Ag_14_ clusterzymes exhibited negligible cytotoxicity to L929 cells at a high concentration of 280 μg/mL, according to CCK-8 (Cell Counting Kit-8) testing data. And the results of colony assays formation also demonstrated that Ag_14_ clusterzymes did not decrease cell reproductive capacity (Fig. 2d). The survival rate of L929 cells treated with Ag_14_ clusterzymes at a concentration of 280 μg/mL before 4 Gy γ-ray irradiation showed significant enhancement compared with those received radiation alone (Fig. 2b). In addition, Colony assays were further performed to assess the cell reproductive capacity of L929 cells treated with or without Ag_14_ clusterzymes followed by 2 Gy γ-ray irradiation. As shown in Fig. 2c, d, compared with the control group, there are only 58.1% colony formations when treated with radiation alone, while 86.9% colony formations are achieved with Ag_14_ clusterzymes incubation before radiation. These results suggested prominent in vitro protection capability of Ag_14_ clusterzymes against γ-ray irradiation. To elucidate the radiation protective mechanism of Ag_14_ clusterzymes, the radiation-induced cellular DNA damage levels with and without Ag_14_ clusterzymes treatment were evaluated by single cell gel electrophoresis (or comet assay). As shown in Fig. 2e, the 4 Gy γ-ray irradiated cells showed long tails in relation to severe cellular DNA damages, while cells treated with Ag_14_ clusterzymes before irradiation showed fewer cell tails and DNA damages. Quantitative analysis of tail moments suggested effective prevention of radiation-induced DNA damages or significant DNA repairs (Fig. 2f).

**Fig. 3:**
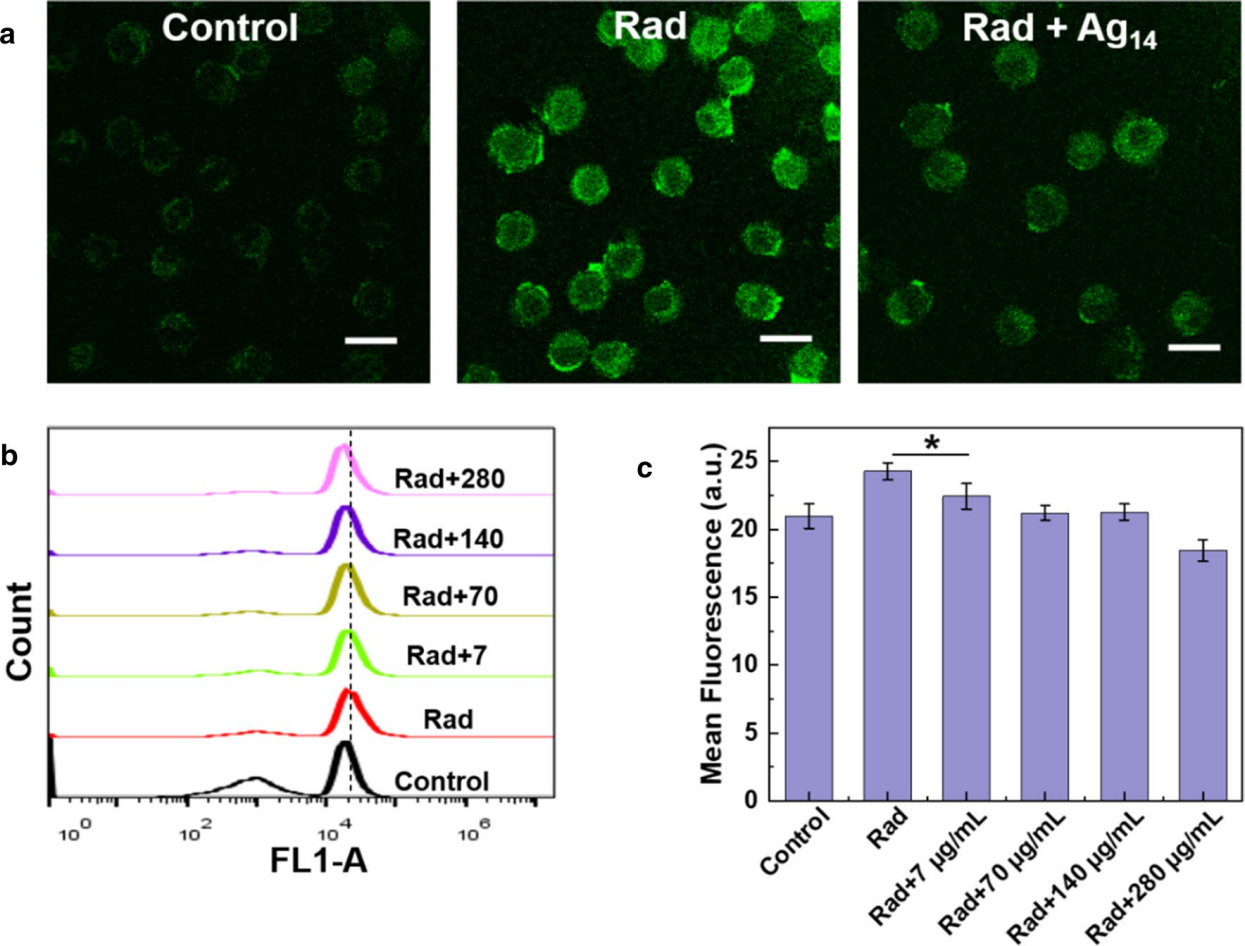
Intracellular ROS levels. **a** Laser confocal fluorescence microscopic images of intracellular ROS levels in L929 cells treated with or without Ag_14_ clusterzymes (280 μg/mL) after 4 Gy radiation. **b**, **c** Quantitative analysis of intracellular ROS levels in L929 cells treated with or without Ag_14_ clusterzymes at various concentrations after 4 Gy radiation using flow cytometry. Scale bars are all 20 μm. p values: ***p < 0.001, **p < 0.01, or *p < 0.05, ANOVA

The oxidative stress modulation of Ag_14_ clusterzymes in vitro was also investigated. Intracellular ROS levels after 4 Gy γ-ray irradiation with or without Ag_14_ clusterzymes treatment were analyzed qualitatively and quantitatively in L929 cells with a ROS probe 2, 7-dichlordihydrofluorescein diacetate (DCFH-DA) (Fig. 3). After reacting with ROS in cell, DCFH molecules were oxidized, and then they emitted bright green fluorescence. Fluorescent images revealed the presence of excessive ROS in cells after γ-ray irradiation. And the treatment of Ag_14_ clusterzymes decreased the fluorescence signals, demonstrating the scavenging ability of Ag_14_ clusterzymes toward ROS in cells (Fig. 3a). Quantitative investigation by flow cytometry showed similar results that the ROS levels induced by γ-ray irradiation in L929 cells were decreased with the concentrations of Ag_14_ clusterzymes increased (Fig. 3b, c).

**Fig. 4:**
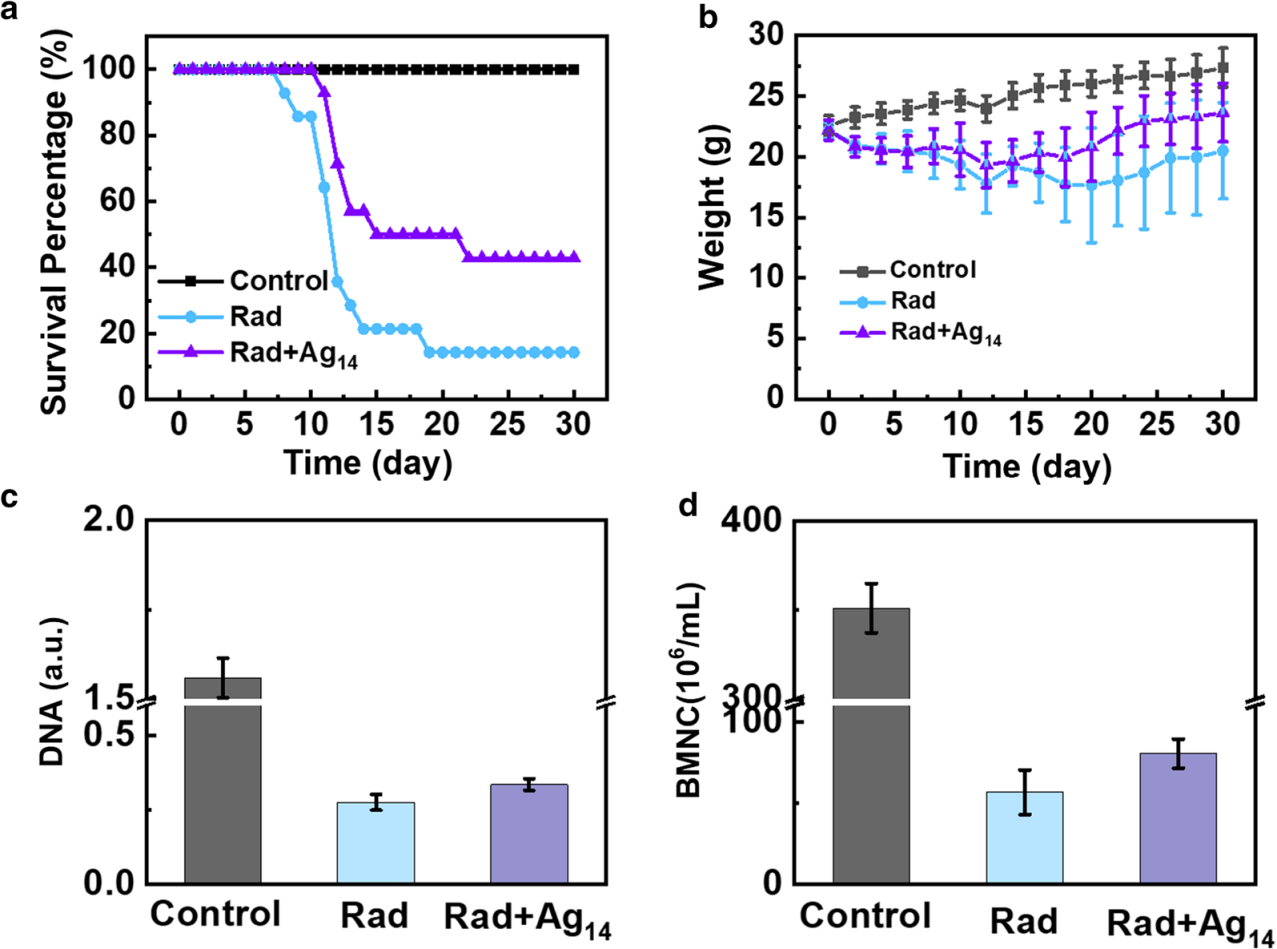
Radioprotection of Ag_14_ clusterzymes in vivo. **a** Survival curves of mice with or without pre-injection of Ag_14_ clusterzymes (1.4 mg/mL, 0.2 mL) after 7 Gy radiation (14 mice per group). **b** Body weight of mice in different groups during 30 days. **c** Bone marrow total DNA content of mice in control group, radiation only group and radiation with pre-injection of Ag_14_ clusterzymes at 7 days, as measured by UV–vis absorption at 268 nm. **d** Counts of bone marrow nucleated cells in the three groups at 7 days

To evaluate the radioprotection effects in vivo, Ag_14_ clusterzymes (1.4 mg/mL, 0.2 mL) was tail intravenously injected into C57 mice 30 min before total body γ-ray irradiation at a dose of 7.0 Gy. The survival fraction of mice was monitored within 30 days, meanwhile the body weight of mice was recorded every 2 days. As shown in Fig. 4a, the survival fraction of irradiated mice without treatment rapidly dropped to 14.3% after 19 days, indicating severe radiation damages caused by γ-ray exposure. However, the survival fraction of Ag_14_ clusterzymes-treated group maintained 50% after 19 days, then 42.9% after 30 days, showing apparent radioprotection effects. Moreover, the body weight of Ag_14_ clusterzymes-treated group recovered better than the untreated group 8 days after radiation (Fig. 4b). These results demonstrated that Ag_14_ clusterzymes could provide efficient protection against in vivo radiotoxicity.

The mechanism study was then conducted to elucidate the radioprotection effect of Ag_14_ clusterzymes in vivo. Amifostine was chosen as positive control. High-energy ionizing radiation can affect the function of the hematopoietic system and inhibit the proliferation and differentiation of bone marrow hematopoietic stem cells. Bone marrow total DNA contents and bone marrow nucleated cells (BMNCs) were used as biomarkers of radiation damage to hematopoietic system. Bone marrow DNA contents of each group were assessed by optical absorption at 268 nm. On the 7th day after radiation, the optical density (OD) value of the Radiation group was 0.275, which was only 17.6% of the control group, while the OD value of Ag_14_ clusterzymes-treated group increased to 0.334 (Fig. 4c). This indicated that γ-ray irradiation caused severe DNA damage, but the damage was alleviated by Ag_14_ clusterzymes treatment. The same trend was found in BMNCs, which sharply decreased to 16.2% of the control group on the 7th day after radiation. BMNCs of the Ag_14_ clusterzymes-treated group displayed mild recovery ability compared with the radiation group (Fig. 4d). The radioprotection effect of Ag_14_ clusterzymes on DNA became more significant with increasing observation time. Both bone marrow total DNA contents and BMNCs showed stronger recovery on 10th day after radiation, and there was a significant difference compared with the radiation group (Additional file 1: Figure S9). When the administration dose was 1.4 mg/mL, amifostine did not show the comparative radioprotective effect as Ag_14_ clusterzymes (Additional file 1: Figure S9). These results demonstrated that Ag_14_ clusterzymes could exert radioprotection effects at a low administration dose by reducing and repairing the radiation damages on hematopoietic systems.

**Fig. 5:**
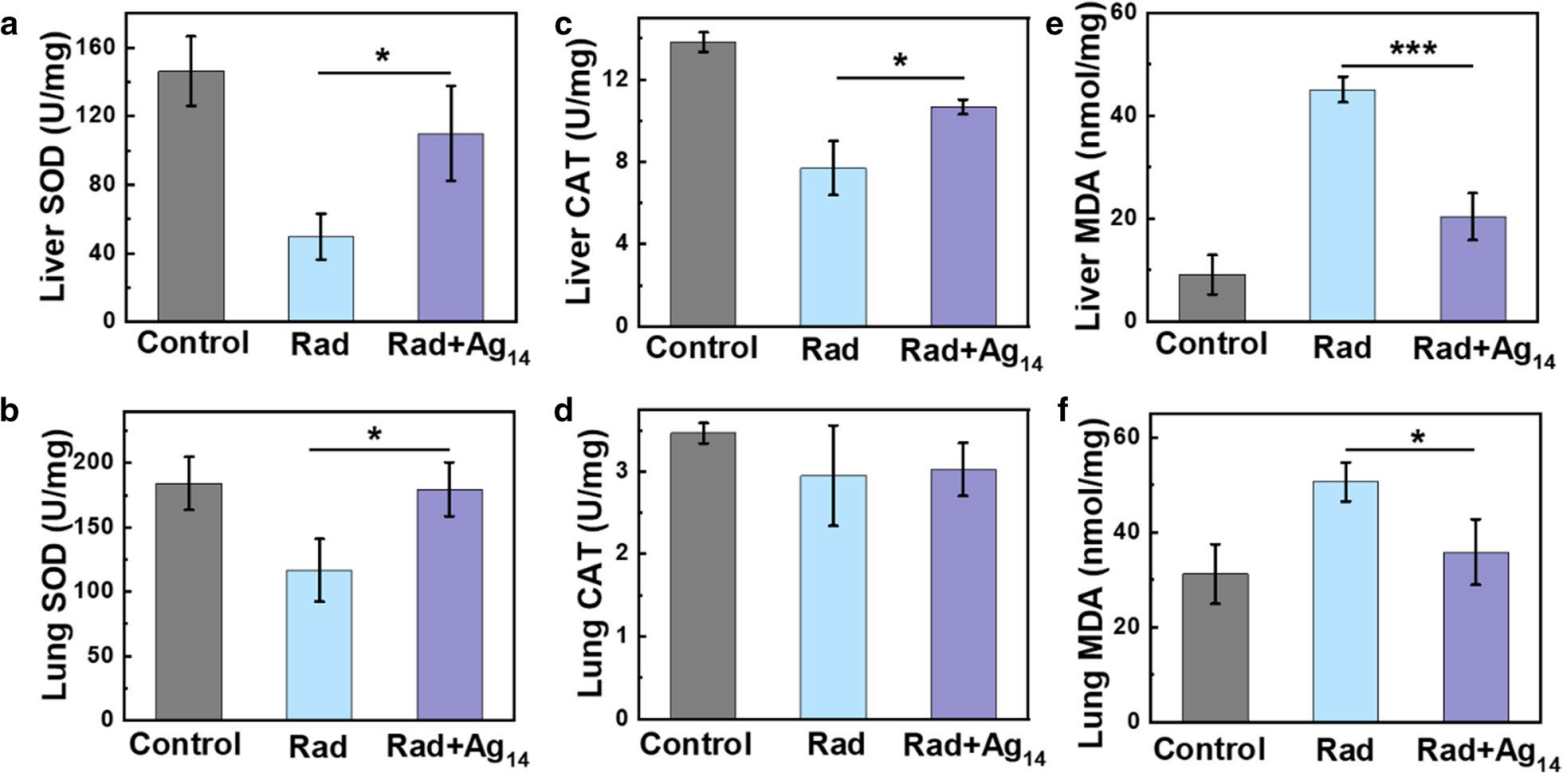
Radioprotection mechanism of Ag_14_ clusterzymes in vivo. Superoxide dismutase (SOD) levels in **a** liver and **b** lung 7 days after 7 Gy radiation. Catalase (CAT) levels in **c** liver and **d** lung. Lipid peroxidation malondialdehyde (MDA) levels in **e** liver and **f** lung. p values: ***p < 0.001, **p < 0.01, or *p < 0.05, ANOVA

In addition, the tissue oxidative stress level was studied to further confirm the radiation protection mechanisms of Ag_14_ clusterzymes. High-energy ionizing radiation can induce severe tissue oxidative stress through ROS generated from water ionization and oxygen participation. Therefore, the endogenous antioxidant enzymes in tissues, such as superoxide dismutase (SOD) and catalase (CAT), were consumed to eliminate excessive ROS. Meanwhile, the lipid peroxidation products increased, such as 3, 4-methylenedioxyamphetamine (MDA). On the 7th day after radiation, the SOD activity of the liver and lung tissues of mice in the radiation group was significantly decreased than that of the control group, while those with Ag_14_ clusterzymes treatment distinctly recovered (Fig. 5a, b). Compared to the control group, the liver and lung SOD activity increased from 34.1 to 75.1% and 63.2% to 97.5%, respectively. Similar results were observed on liver CAT activities in each group. The activity values decreased from 13.8 to 7.7 U/mg after radiation, but recovered to 10.7 U/mg with the treatment of Ag_14_ clusterzymes (Fig. 5c). However, there was no significant difference in CAT activity in the lung tissue of each group on the 7th day after radiation, which may be due to the inaccurate detection time selected (Fig. 5d). As shown in Fig. 5e, f the liver and lung tissues MDA contents of the radiation group increased significantly compared with the control group, indicating severe lipid peroxidation of major organs induced by γ-ray irradiation. However, injection of Ag_14_ clusterzymes before radiation could recover the MDA content in liver and lung tissues back to the normal levels on the 7th day after radiation. These results demonstrated that Ag_14_ clusterzymes could exert radioprotection effect by reducing the consumption of endogenous antioxidant enzymes and repairing tissue oxidative stress in vivo induced by irradiation exposure.

We also explored the effect of Ag_14_ clusterzymes on in vivo tumor radiotherapy using mice bearing 4T1 tumors. As shown in Additional file 1: Figure S10, Ag_14_ clusterzymes did not increase radioresistance in the tumors. There was no significant difference in tumor size (volume and weight) between the Ag_14_ clusterzymes-treated group and the γ-ray irradiation group. We present a hypothesis on how Ag_14_ clusterzymes can protect normal tissues from radiation damage without causing tumor radiation resistance. First, during tumor radiotherapy, the tumor region was locally irradiated by γ-ray irradiation. Thus, the radiation dose received by tumor tissue is much greater than that of the normal tissues. The direct killing effects of •OH on tumor cells are dominant. Furthermore, tumor tissues proliferate rapidly and tumor cells have a more relaxed chromatin structure compared with normal tissues. Therefore, they are more susceptible to radiation damage than normal tissues [1, 23, 29].

**Fig. 6:**
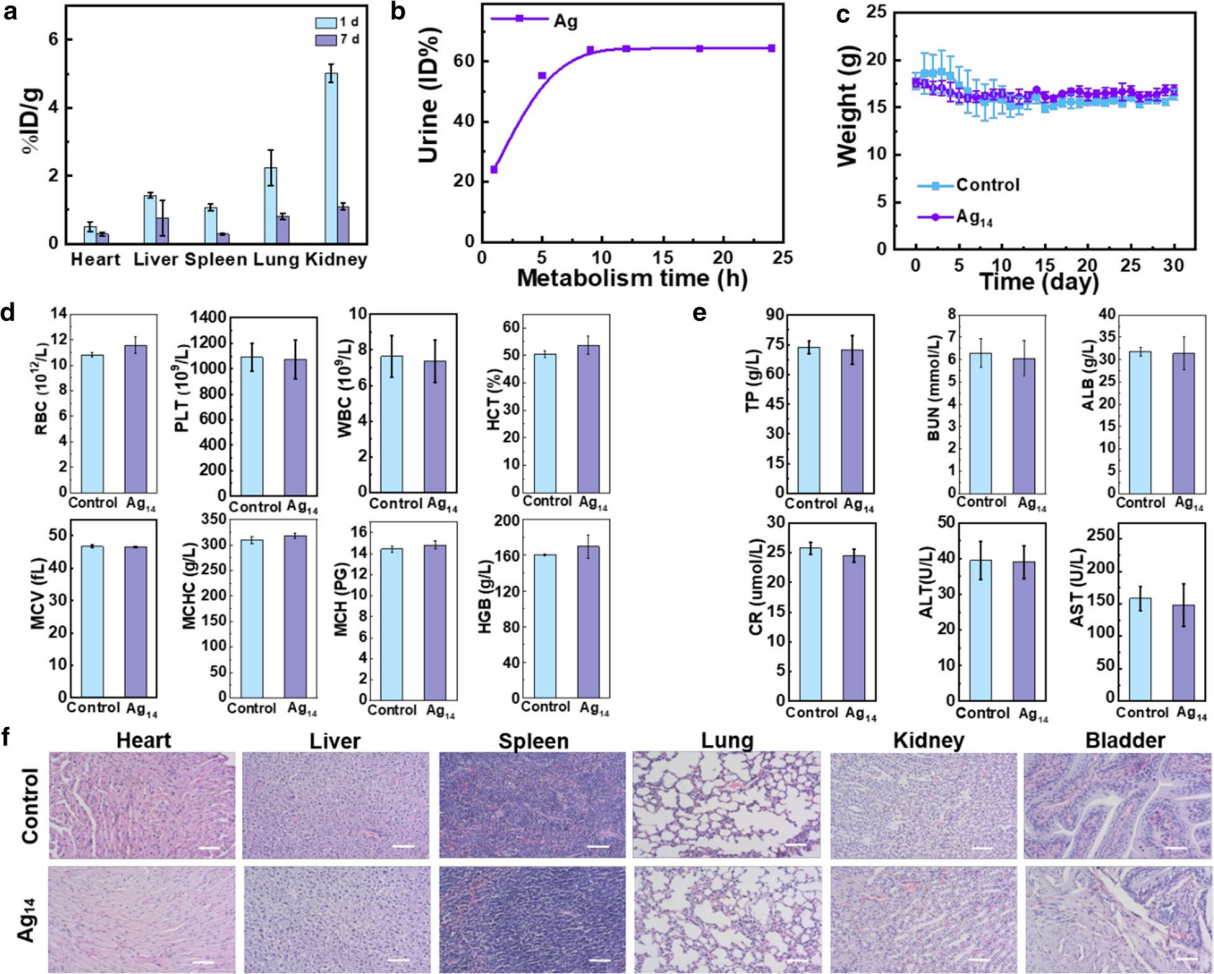
Biodistribution and in vivo toxicities. **a** Biodistribution of Ag_14_ clusterzymes in major organs at 1 and 7 days post intravenously injection of Ag_14_ clusterzymes. **b** Cumulative urine excretion of Ag_14_ clusterzymes at different time points. **c** Body weight of mice untreated mice and mice treated with Ag_14_ clusterzymes 30 days post-injection. **d** Hematological indicators and **e** serum biochemical indexes of control group and experimental group treated with Ag_14_ clusterzymes 30 days post-injection. **f** Pathological evaluation of control group and experimental group treated with Ag_14_ clusterzymes 30 days post-injection. Scale bars are all 50 μm

Furthermore, the pharmacokinetics, biodistribution, and in vivo toxicity were investigated. The half-life of Ag_14_ clusterzymes in blood was about 46 min (Additional file 1: Figure S11). The kidney showed the highest uptake at 1 day post injection and urine excretion test showed that 64.3% of Ag_14_ clusterzymes were excreted through renal clearance within 24 h after intravenous injection (Fig. 6a, b). Ag_14_ clusterzymes were rarely accumulated in the main organs at 7 day post injection, indicating that Ag_14_ clusterzymes could be excreted out of the body through urine without causing long-term toxic effect (Fig. 6a). During the 30 days observation period, Ag_14_ clusterzymes did not cause any abnormal effect on body weight and behavior of mice included (Fig. 6c). Hematological indicators, such as white blood cell (WBC), red blood cell (RBC), hemoglobin (HGB) and platelets (PLT), and serum biochemical indexes, such as alanine transaminase (ALT), aspartate aminotransferase (AST), blood urine nitrogen (BUN) and serum creatinine (CERA), were examined and no statistical difference was observed compared with the control group, suggesting negligible hemotoxic, hepatotoxicity and nephrotoxicity (Fig. 6d, e). Main organs including heart, liver, spleen, lung, kidney and bladder were collected for pathological analysis by hematoxylin and eosin (H&E) staining. As shown in Fig. 6f, no apparent damage was observed in all organs, especially the spleen and kidney. These results demonstrated that the water soluble Ag_14_ clusterzymes with ultrasmall size could be excreted out of the body quickly without causing long-term toxicity in vivo.

## Discussion

In this study, we investigated the potential of atomically accurate Ag_14_ clusterzymes as a new type of radioprotectants for the first time. Different from other Ag nanomaterials, which have been employed for antimicrobial applications and cancer treatment, the as-prepared Ag_14_ clusterzymes showed superoxide dismutase (SOD)-like activity, antioxidant capacity, especially strong •OH scavenging ability. In vitro cell experiments showed that Ag_14_ clusterzymes could improve cell viability, eliminate ROS and prevent DNA damages in cells after γ-ray irradiation. In vivo experiments showed that Ag_14_ clusterzymes could improve the overall survival rate of the irradiated mice, protect hematological system and bone marrow DNA against γ-ray irradiation. In addition, Ag_14_ clusterzymes could relieve the oxidative stress in vivo through regaining endogenous reductive enzymes, including SOD and CAT, and removing excessive lipid peroxidation products induced by γ-ray irradiation, such as MDA. In addition, Ag_14_ clusterzymes did not increase radioresistance of 4T1 tumors. Compared with small molecule radioprotectants, the as-prepared Ag_14_ clusterzymes have appropriate blood elimination half-life. And due to the ultrasmall size and water soluble, Ag_14_ clusterzymes could be rapidly excreted out of the body through kidneys and did not cause any toxicological responses. Importantly, for atomically precise molecular structure, the catalytic activity and selectivity of the Ag_14_ clusterzymes can be regulated at molecular levels.

Furthermore, although we have studied the fluorescence properties of Ag_14_ clusterzymes in this work, these properties in biomedicine have not been explored. Yang et al. [34], explored the application of Ag_14_ clusters in cancer cell imaging, but they did not conduct in-depth research in vivo, so the application of Ag_14_ clusterzymes in bio-imaging is still worthy of further exploration. We could adjust the catalytic and optical properties of Ag_14_ clusterzymes at the molecular level by changing the composition and the ratio of metal atoms and ligand molecules, so that could further expand the applications of Ag_14_ clusterzymes in disease diagnosis and treatment.

## Conclusions

In summary, atomically precise, ultrasmall and water soluble Ag_14_ clusterzymes with SOD-like activity were successfully developed and proved to be effective both in vitro and in vivo for radioprotection. Furthermore, with atomically precise molecular structure, Ag_14_ clusterzymes, on aspect of the catalytic and optical properties, may be improved by structure optimization on atom-scale level for other applications in disease diagnosis and treatment.

## Methods

### Materials

Silver nitrate (AgNO_3_) was purchased from Beijing TanMo Quality Testing Technology (China); l-glutathione reduced (GSH, ≥ 97%), sodium borohydride (NaBH_4_, ≥ 97%), and sodium hydroxide (NaOH, ≥ 97%) were purchased from Tianjin heowns Biochemical Technology (China); Salicylic acid (C_7_H_6_O_3_, ≥ 99.5%) and ferrous sulfate (FeSO_4_, ≥ 98%) were purchased from Shanghai Macklin Biochemical (China); Ultrapure Millipore water (H_2_O, 18.2 MΩ) was used in the whole study.

### Preparation of Ag_14_ clusterzymes

Ag_14_ clusterzymes were synthesized and purified according to a literature report [35]. In a typical synthesis, aqueous solution of GSH (150 μL, 50 mM) and AgNO_3_ solution (125 μL, 20 mM) were added to ultrapure water (4.85 mL) under vigorous stirring condition to form a white flocculent precipitation. Then freshly prepared NaBH_4_ solution (50 μL, prepared by adding 43 mg NaBH_4_ to 10 mL 0.2 M NaOH solution) was introduced into the mixture to obtain a deep-red solution in 5 min. After incubated at room temperature for 3 h, another 50 μL freshly prepared NaBH_4_ solution was added and the mixture changed to brown 15 min later. For further purification, the raw solution was traveled through a desalting column (PD-10, GE Healthcare) to get rid of small categories (e.g., free ligands and salts). The as-purified Ag_14_ clusterzymes aqueous solution was collected for characterization and further application.

### Characterization

UV–vis absorption spectra was tested on a Shimadzu UV-1800 spectrometer. Fluorescence spectra was recorded on a Fluorescence spectrophotometer (HITACHI F-4600 (Japan)), the excitation wavelength was 480 nm. ESI–MS were acquired on Bruker microTOF-Q system. Transmission electron microscopy (TEM) was performed on a JEOL JEM 2100F microscope operating at 200 kV. ICP-MS was tested with Agilent 7500 CE (Agilent Technologies, Waldbronn, Germany). Native PAGE was carried out on a Bio-Rad Mini-PROTEAN^®^ Tetra Cell or PROTEAN^®^ II xi Cell system. Stacking and resolving gels were prepared from 4 and 15 wt% acrylamide monomers, respectively. Negative electrophoretic buffer containing Tricine. PD-10 desalting columns (GE Healthcare UK Ltd) containing 8.3 mL of SephadexTM G-25 medium with a molecular weight exclusion limit of 5000 Da.

### Hydroxyl radical scavenging ability test

Salicylic acid (SA) could be hydroxylated by •OH generated from ordinary Fenton reaction to produce purple product, which has distinct absorbance at 510 nm. In a typical •OH clearance test, 200 μL H_2_O_2_ (50 mM) was added to a solution mixed with 200 μL SA solution (10 mM, water:ethanol = 9:1), 200 μL FeSO_4_ (10 mM), 100 μL Ag_14_ clusterzymes (1.4 mg/mL), 200 μL ultrapure water and 100 μL PBS (0.01 M). After incubation for 30 min at room temperature, the UV–vis absorption spectra was recorded on a Shimadzu UV-1800 spectrometer, the absorbance at 510 nm was recorded on a microplate reader.

### SOD-like activity test

The SOD-like activity of Ag_14_ clusterzymes was measured in accordance with the manufacturer’s instructions in SOD assay kit (Beyotime, S0101M), and the SOD-like activity of a series concentrations of Ag_14_ clusterzymes was expressed as the percentage inhibition of WST-8 reaction with superoxide anion.

### ESR test

ESR spectra of the BMPO spin-adducts were measured on a Bruker EMX spectrometer (Germany), X-band ~ 9.8 GHz, 3510 G central field, 200 scan range, 20 mW microwave power, 1 G modulation amplitude, 20.48 ms time constant and 20.48 ms conversion time.

#### •OH scavenging test

1 mM H_2_O_2_ solution was prepared with phosphate buffer (pH = 7.4, 0.01 M) and BMPO (25 mM) was used as spin trap for •OH. Then, •OH was generated by UV-laser irradiation for 5 min. After irradiation, the sample was transferred to a standard cavity ESR flat cell. The scavenging process of •OH was determined by testing the signal intensity change of peaks before and after the addition of Ag_14_ clusterzymes.

#### O_2_^•−^ scavenging test

KO_2_/DMSO (20 mM) solution and 18-crown-6 (3.5 mM) were used as the generation source and stabilizer of O_2_^•−^. BMPO (25 mM) was used as spin trap for O_2_^•–^ and its spin adduct (BMPO/OOH•) presented six peaks under ESR spectrometer. The BMPO/KO_2_ sample was mixed for 10 s and transferred to a standard cavity ESR flat cell. The scavenging process of O_2_^•−^ was determined by monitoring the signal intensity change of peaks before and after the addition of Ag_14_ clusterzymes.

### Antioxidation capacity test

The anti-oxidation capacity test of Ag_14_ clusterzymes was carried out at room temperature by employing a commercial colorimetric Total Antioxidation Capacity Assay Kit (Beyotime, S0121). The assay was conducted in accordance with the manufacturer’s instructions. The time-dependent anti-oxidation activity of series concentrations of Ag_14_ clusterzymes were represented by the absorbance at 414 nm.

### Cytotoxicity

L929 mouse fibroblast cells were cultured in DMEM cell culture medium containing 10% fetal bovine serum (FBS) at 37 °C, 5% CO_2_. For cytotoxicity test, L929 cells were cultured in 96-well plates (4 × 10^3^/well) in 100 μL DMEM with 10% FBS for 24 h and incubated with a series concentrations of Ag_14_ clusterzymes (from 0 to 280 μg/mL) for another 24 h. Then CCK-8 (Cell Counting Kit-8) assay was employed to investigate the relative cell cytotoxicity following the manufacturer’s instructions (Beyotime, C0037).

### Cellular uptake

L929 cells were seeded on a 6-well plate at 1 × 10^6^ cells per well and cultured in DMEM cell culture medium containing 10% fetal bovine serum (FBS) at 37 °C, 5% CO_2_ for 24 h. Then the treated groups were incubated with Ag_14_ clusterzymes (280 μg/mL) for 1 h. After that, cell media were discarded and the cells were washed with PBS for three times, then it was trypsinized and resuspended. The cells were counted before sent for testing Ag contents using inductively coupled plasma mass spectrometry (ICP-MS).

### Cell survival

L929 cells were seeded in two 96-well plates (4 × 10^3^/well) in DMEM with 10% FBS for 24 h and then incubated with Ag_14_ clusterzymes (280 μg/mL) for 1 h followed by γ-ray irradiation at a dose of 0 Gy and 4 Gy. After irradiation, the cells were incubated at 37 °C, 5% CO_2_ for another 24 h and then CCK-8 assay was carried out to investigate the relative cell viabilities following the manufacturer’s instructions (Beyotime, C0037).

### Colony assay

L929 cells were cultured in two 6-well plates in DMEM with 10% FBS for 24 h and incubated with Ag_14_ clusterzymes (280 μg/mL) for 1 h followed by γ-ray irradiation at a dose of 0 Gy and 2 Gy. Cells were incubated at 37 °C, 5% CO_2_ for further 5 h. Then the medium was replaced by fresh DMEM with 10% FBS and the cells were incubated for 9–12 days. Once colony formations observed were greater than 50, the culture medium was discarded, and the plates were rinsed twice with PBS. After that, 4% paraformaldehyde solution (500 μL/well) was added to fix the cells for 10 min, and then the cells were stained with crystal violet for 10 min after immobilization. At last, the crystal violet was discarded and the colony formations were counted manually after rinsed with PBS.

### Comet assay

Comet assay was carried out by employing a commercial DNA damge detection kit (SCGE) from KeyGEN BioTECH (China). The cells were prepared as follows before gel electrophoresis: L929 cells were cultured in two 6-well plates (3 × 10^5^/well) in DMEM with 10% FBS for 24 h and incubated with Ag_14_ clusterzymes (280 μg/mL) for 1 h followed by γ-ray irradiation at a dose of 0 Gy and 4 Gy. Cells were incubated at 37 °C, 5% CO_2_ for further 1 h. The medium was then removed and the cells were washed, trypsinized, centrifuged and resuspended in PBS to get a cell density of 1 × 10^6^/mL. Then the assay was performed according to the manufacturer’s instructions. DNA damages were analyzed by fluorescent microscope (ZEISSVert.A1), and the tail moments were assessed with Comet Assay Software.

### Intracellular ROS measurements

L929 cells were cultured in 6-well plates (1 × 10^5^/well) in DMEM with 10% FBS for 24 h and incubated with Ag_14_ clusterzymes (280 μg/mL) for 1 h followed by γ-ray irradiation at a dose of 4 Gy. After irradiation, the cells were incubated at 37 °C for another 24 h and then incubated with 1 mL 2,7-dichlorodihydrofluorescein diacetate (DCFH-DA, 5 μM) for 20 min after removing the medium. Laser confocal fluorescence microscope was applied to observe the fluorescence intensity of each group. The cells were trypsinized and resuspended in PBS for quantitative investigation of the fluorescence intensity by flow cytometry.

### In vivo radiation protection

All animal-related experiments protocols were reviewed and permitted by the Institutional Animal Care and Use Committee (IACUC). All animals were purchased, raised and dealt with according to procedures approved by the Institute of Radiology Medicine of the Chinese Academy of Medical Sciences. 38 male C57 mice aged 6–8 weeks, weighing 18–22 g were randomly divided into three groups: 1, control group (10 mice, with 0.2 mL PBS injection only); 2, radiation group (14 mice, 0.2 mL PBS plus 7.0 Gy γ-ray irradiation); 3, Ag_14_ clusterzymes + radiation group (14 mice, Ag_14_ clusterzymes (0.2 mL, 1.4 mg/mL) plus 7.0 Gy γ-ray irradiation). 30 min after tail intravenous injection of PBS or Ag clusterzymes, mice in group 2 and 3 received total body γ-ray exposure at a dose of 7 Gy. All mice were observed for 30 days and the weight was recorded every two days at the same time. Mice that were still alive after 30 days were killed by cervical dislocation.

### Bone marrow DNA and BMNC evaluations

To analyze bone marrow DNA contents and bone marrow nucleated cells (BMNCs) of mice under γ-ray irradiation, 3 mice of three experiment groups were sacrificed at 7 days and bilateral femurs of all mice were extracted(major organs were also collected for tissues oxidative stress analysis). To estimate the total DNA contents in bone marrow, the femurs were washed repeatedly by quantified calcium chloride solution (10 mL, 5 mM), then the suspensions were placed for 30 min at 4 °C and centrifuged for 15 min at 2500 rpm. After discarding the supernatants, the precipitates were mixed with perchlorate (5 mL, 0.2 M) and then dealt with water bath at 90 °C for 15 min. The suspensions were filtrated with 0.22 μm Millex-GP membrane filters after cooled to room temperature and the absorbance of the filtrates was measured at 268 nm with UV–vis spectrophotometer. The other femur of the mouse was obtained similarly and washed with 1 mL PBS until the femur turned white. The suspensions were then filtrated with a 300-mesh gauze and the BMNCs in the filtrate were counted by hemocytometer.

### Oxidative stress

The livers and lungs for superoxide dismutase (SOD), Malondialdehyde (MDA) and catalase (CAT) analyses were also from the 7 days sacrificed mice. The tips of liver acquired about 0.2 g and lung respectively immersed in saline solution (2 mL) and ground to 10% tissue homogenates. Then the homogenates were kept on ice for 2 h and centrifuged at 4 °C, 2500 rpm for 10 min. The supernatants were collected and further diluted as needed, respectively. The protein content, SOD, MDA and CAT levels in the tissues were quantified by employing BCA Protein Assay Kit (P0011), Total Superoxide Dismutase Assay Kits with NBT (S0109), Lipid Peroxidation MDA Assay Kits (S0131S) and Catalase Assay Kit (S0051) from Beyotime Biotechnology (China) following manufacturer’s instructions, respectively.

### Effect on in vivo tumor radiotherapy

To prepare the tumor model, murine breast cancer 4T1 cells (1.8 × 10^6^) suspended in 200 μL PBS were subcutaneously injected into the back of each Balb/c mouse. Mice bearing 4T1 tumors were randomly divided into four groups (five mice per group): 1, control group with PBS injection; 2, Ag_14_ clusterzymes injection group; 3, PBS injection + RT group; 4, Ag_14_ clusterzymes injection + RT group. The dose for intravenous injection was 0.2 mL per mouse, and the concentration of Ag_14_ clusterzymes was 1.4 mg/mL. When the tumor volume reached ~ 60 mm^3^, mice in group 3 and 4 received a 8 Gy γ-ray local radiation for tumor on Day 0 after 30 min post-injection of PBS or Ag_14_ clusterzymes. The lengths and widths of tumors were measured by a vernier caliper every 2 days. The tumor volume was calculated by an equation of volume = width^2^ × length/2. The mice from all groups were sacrificed and tumors were excised for weighting and photograph at 12th day after various treatment.

### Pharmacokinetics, biodistribution, and in vivo toxicity

C57 mice were injected intravenously with Ag_14_ clusterzymes (1.4 mg/mL, 0.2 mL) to evaluate pharmacokinetics and biodistribution by testing Ag contents using ICP-MS. Blood from three mice was drawn at time points of 5 min, 15 min, 0.5, 1, 6, 12, 24, and 48 h. The main organs were collected at 1 and 7 day after administration. 10 Female Balb/c mice weighing 18–22 g were randomly divided into two groups: control and Ag_14_ clusterzymes treated groups (n = 5) for in vivo toxicity and excretion analyses. The treated group was tail intravenously injected with Ag_14_ clusterzymes (1.4 mg/mL, 0.2 mL) while the control group was with 0.2 mL PBS. All mice were observed for 30 days and precisely weighted every 2 days at the same time. The mice were sacrificed at 30 days and the blood samples by eyeball extirpating were obtained for hematology and biochemistry studies. 20 μL of the blood sample was dripped into potassium EDTA collection tube for hematology analysis. The rest of the blood (about 500 μL) were kept at 4 °C overnight and then centrifuged at 6000 rpm 4 °C for 5 min to collect the serum for biochemistry evaluation. Main organs of two mice including heart, liver, spleen, lung, kidney and bladder were collected and stained by hematoxylin and eosin (H&E) for pathological analyses.

### Statistical analysis

All data were presented as average ± standard deviations (SD). Analysis of variance (ANOVA) statistics were carried out, and p-values less than 0.05 were regarded as statistically significant.

## Supplementary Information

**Additional file 1. **Additional Figures S1–S11.

## Data Availability

Additional file is available online or by request.

## References

[1] Tubiana M (1992). The role of local treatment in the cure of cancer. Eur J Cancer.

[2] Song G, Cheng L, Chao Y, Yang K, Liu Z (2017). Emerging nanotechnology and advanced materials for cancer radiation therapy. Adv Mater.

[3] Ahmad SS, Duke S, Jena R, Williams MV, Burnet NG (2012). Advances in radiotherapy. BMJ.

[4] Liu J, Liu C, Yue J (2021). Radiotherapy and the gut microbiome: facts and fiction. Radiat Oncol.

[5] Palata O, Hradilova Podzimkova N, Nedvedova E, Umprecht A, Sadilkova L, Palova Jelinkova L, Spisek R, Adkins I (2019). Radiotherapy in combination with cytokine treatment. Front Oncol.

[6] Bai L, Jiang F, Wang R, Lee C, Wang H, Zhang W, Jiang W, Li D, Ji B, Li Z (2020). Ultrathin gold nanowires to enhance radiation therapy. J Nanobiotechnol.

[7] Newhauser WD, Durante M (2011). Assessing the risk of second malignancies after modern radiotherapy. Nat Rev Cancer.

[8] Rabender CS, Mezzaroma E, Yakovlev VA, Mauro AG, Bonaventura A, Abbate A, Mikkelsen RB (2021). Mitigation of radiation-induced lung and heart injuries in mice by oral sepiapterin after irradiation. Radiat Res.

[9] Voshart DC, Wiedemann J, van Luijk P, Barazzuol L (2021). Regional responses in radiation-induced normal tissue damage. Cancers (Basel).

[10] Bentzen SM (2006). Preventing or reducing late side effects of radiation therapy: radiobiology meets molecular pathology. Nat Rev Cancer.

[11] Helissey C, Cavallero S, Brossard C, Dusaud M, Chargari C, Francois S (2020). Chronic inflammation and radiation-induced cystitis: molecular background and therapeutic perspectives. Cells.

[12] Lauber K, Ernst A, Orth M, Herrmann M, Belka C (2012). Dying cell clearance and its impact on the outcome of tumor radiotherapy. Front Oncol.

[13] Chandra J, Samali A, Orrenius S (2000). Triggering and modulation of apoptosis by oxidative stress. Free Radic Biol Med.

[14] Ryter SW, Kim HP, Hoetzel A, Park JW, Nakahira K, Wang X, Choi AM (2007). Mechanisms of cell death in oxidative stress. Antioxid Redox Signal.

[15] De Ruysscher D, Niedermann G, Burnet NG, Siva S, Lee AWM, Hegi-Johnson F (2019). Radiotherapy toxicity. Nat Rev Dis Primers.

[16] Mittal M, Siddiqui MR, Tran K, Reddy SP, Malik AB (2014). Reactive oxygen species in inflammation and tissue injury. Antioxid Redox Signal.

[17] Yao J, Cheng Y, Zhou M, Zhao S, Lin S, Wang X, Wu J, Li S, Wei H (2018). ROS scavenging Mn_3_O_4_ nanozymes for in vivo anti-inflammation. Chem Sci.

[18] Griendling KK, FitzGerald GA (2003). Oxidative stress and cardiovascular injury: part I: basic mechanisms and in vivo monitoring of ROS. Circulation.

[19] Cakmak G, Miller LM, Zorlu F, Severcan F (2012). Amifostine, a radioprotectant agent, protects rat brain tissue lipids against ionizing radiation induced damage: an FTIR microspectroscopic imaging study. Arch Biochem Biophys.

[20] King M, Joseph S, Albert A, Thomas TV, Nittala MR, Woods WC, Vijayakumar S, Packianathan S (2020). Use of amifostine for cytoprotection during radiation therapy: a review. Oncology.

[21] Fan S, Meng Q, Xu J, Jiao Y, Zhao L, Zhang X, Sarkar FH, Brown ML, Dritschilo A, Rosen EM (2013). DIM (3,3'-diindolylmethane) confers protection against ionizing radiation by a unique mechanism. Proc Natl Acad Sci USA.

[22] Grdina DJ, Murley JS, Kataoka Y (2002). Radioprotectants: current status and new directions. Oncology.

[23] Tarnuzzer RW, Colon J, Patil S, Seal S (2005). Vacancy engineered ceria nanostructures for protection from radiation-induced cellular damage. Nano Lett.

[24] Colon J, Hsieh N, Ferguson A, Kupelian P, Seal S, Jenkins DW, Baker CH (2010). Cerium oxide nanoparticles protect gastrointestinal epithelium from radiation-induced damage by reduction of reactive oxygen species and upregulation of superoxide dismutase 2. Nanomedicine.

[25] Xie J, Wang N, Dong X, Wang C, Du Z, Mei L, Yong Y, Huang C, Li Y, Gu Z, Zhao Y (2019). Graphdiyne nanoparticles with high free radical scavenging activity for radiation protection. ACS Appl Mater Interfaces.

[26] Zhang XD, Zhang J, Wang J, Yang J, Chen J, Shen X, Deng J, Deng D, Long W, Sun YM (2016). Highly catalytic nanodots with renal clearance for radiation protection. ACS Nano.

[27] Wang JY, Mu X, Li Y, Xu F, Long W, Yang J, Bian P, Chen J, Ouyang L, Liu H (2018). Hollow ptpdrh nanocubes with enhanced catalytic activities for in vivo clearance of radiation-induced ROS via surface-mediated bond breaking. Small.

[28] Bai X, Wang J, Mu X, Yang J, Liu H, Xu F, Jing Y, Liu L, Xue X, Dai H (2017). Ultrasmall ws_2_ quantum dots with visible fluorescence for protection of cells and animal models from radiation-induced damages. ACS Biomater Sci Eng.

[29] Ren X, Huo M, Wang M, Lin H, Zhang X, Yin J, Chen Y, Chen H (2019). Highly catalytic niobium carbide (mxene) promotes hematopoietic recovery after radiation by free radical scavenging. ACS Nano.

[30] Jiang X, Du B, Huang Y, Zheng J (2018). Ultrasmall noble metal nanoparticles: breakthroughs and biomedical implications. Nano Today.

[31] Kang X, Chong H, Zhu M (2018). Au_25_(SR)_18_: the captain of the great nanocluster ship. Nanoscale.

[32] Liu H, Li Y, Sun S, Xin Q, Liu S, Mu X, Yuan X, Chen K, Wang H, Varga K (2021). Catalytically potent and selective clusterzymes for modulation of neuroinflammation through single-atom substitutions. Nat Commun.

[33] Sun S, Liu H, Xin Q, Chen K, Ma H, Liu S, Mu X, Hao W, Liu S, Gao Y (2021). Atomic engineering of clusterzyme for relieving acute neuroinflammation through lattice expansion. Nano Lett.

[34] Yang J, Xia N, Wang X, Liu X, Xu A, Wu Z, Luo Z (2015). One-pot one-cluster synthesis of fluorescent and bio-compatible Ag_14_ nanoclusters for cancer cell imaging. Nanoscale.

[35] Yuan X, Yao Q, Yu Y, Luo Z, Dou X, Xie J (2013). Traveling through the desalting column spontaneously transforms thiolated Ag nanoclusters from nonluminescent to highly luminescent. J Phys Chem Lett.

[36] Rai M, Yadav A, Gade A (2009). Silver nanoparticles as a new generation of antimicrobials. Biotechnol Adv.

[37] Adebayo-Tayo BC, Ekundayo-Obaba O, Falodun OI (2020). Antimicrobial potential of bioactive metabolites and silver nanoparticles from bacillus spp. and of some antibiotics against multidrug resistant salmonella spp.. Turk J Pharm Sci.

[38] Tang J, Shi H, Ma G, Luo L, Tang Z (2020). Ultrasmall Au and Ag nanoclusters for biomedical applications: a review. Front Bioeng Biotechnol.

[39] Liu M, He D, Yang T, Liu W, Mao L, Zhu Y, Wu J, Luo G, Deng J (2018). An efficient antimicrobial depot for infectious site-targeted chemo-photothermal therapy. J Nanobiotechnol.

[40] Yuan X, Setyawati MI, Tan AS, Ong CN, Leong DT, Xie J (2013). Highly luminescent silver nanoclusters with tunable emissions: cyclic reduction–decomposition synthesis and antimicrobial properties. NPG Asia Mater.

[41] Wang Z, Fang Y, Zhou X, Li Z, Zhu H, Du F, Yuan X, Yao Q, Xie J (2020). Embedding ultrasmall Ag nanoclusters in Luria-Bertani extract via light irradiation for enhanced antibacterial activity. Nano Res.

[42] Tian R, Xu J, Luo Q, Hou C, Liu J (2020). Rational design and biological application of antioxidant nanozymes. Front Chem.

[43] Akhtar MJ, Ahamed M, Alhadlaq HA, Khan MAM, Alrokayan SA (2015). Glutathione replenishing potential of CeO(2) nanoparticles in human breast and fibrosarcoma cells. J Colloid Interface Sci.

[44] Liang M, Yan X (2019). Nanozymes: from new concepts, mechanisms, and standards to applications. Acc Chem Res.

