# Supplementary material for: Atomically precise silver clusterzymes protect mice from radiation damages
Source: J Nanobiotechnology. 2021 Nov 19;19:377. doi: 10.1186/s12951-021-01054-5 (PMC8605545; doi:10.1186/s12951-021-01054-5)
Supplement: Supplementary file 1 — Additional file 1. Additional Figures S1–S11. [file 12951_2021_1054_MOESM1_ESM.docx]

Additional Information

Atomically precise silver clusterzymes protect mice from radiation damages

Jiao Guo^1†^, Haiyu Yang^1†^, Ya Liu^1†^, Wei Liu^1^, Ruiying Zhao^3^, He Li^1^, Wei Long^1^*, Wenqing Xu^1^*, Meili Guo^3^*, Xiaodong Zhang^2^*

^1^ Tianjin Key Laboratory of Radiation Medicine and Molecular Nuclear Medicine, Institute of Radiation Medicine, Chinese Academy of Medical Sciences and Peking Union Medical College, 300192Tianjin, China

^2^ Department of Physics and Tianjin Key Laboratory of Low Dimensional Materials Physics and Preparing Technology, School of Sciences, Tianjin University,300350 Tianjin, China

^3^ Department of Physics, School of Science, Tianjin Chengjian University, Tianjin 300384, China

*Correspondence: longway@irm-cams.ac.cn, xuwenqing@irm-cams.ac.cn, meiliguo314@163.com (Meili Guo), xiaodongzhang@tju.edu.cn

^†^Jiao Guo, Haiyu Yang and Ya Liu contributed equally to this work


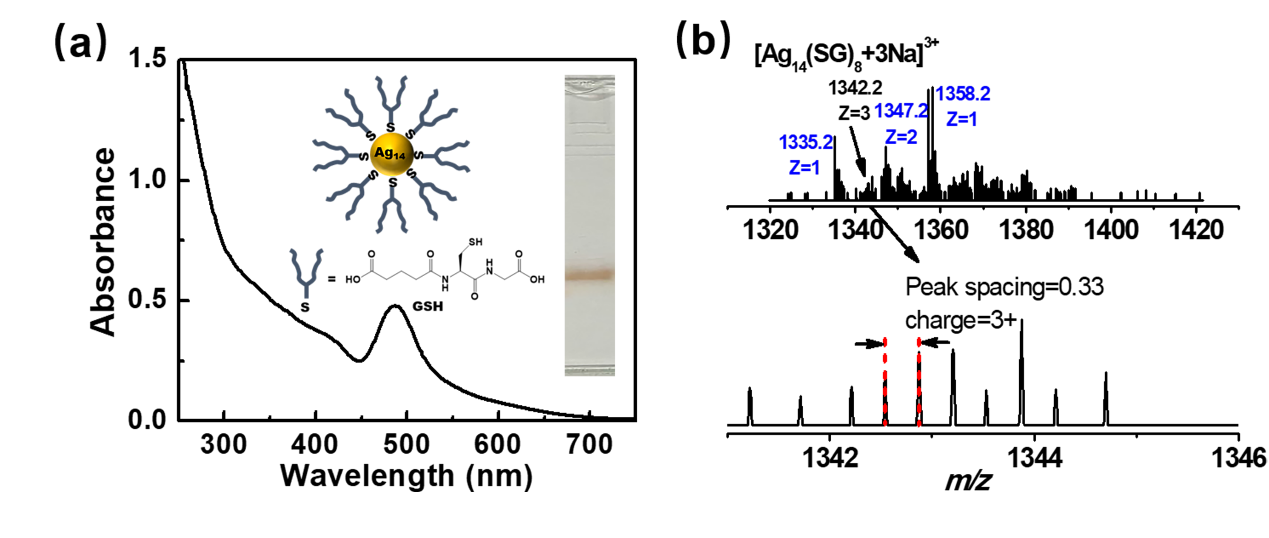


**Figure S1.** (a) The absorption spectra of the Ag_14_ clusterzymes in water. Inset: schematic illustration and photographs of the PAGE gel. (b) ESI mass spectra (in positive ion mode) of the the Ag_14_ clusterzymes.


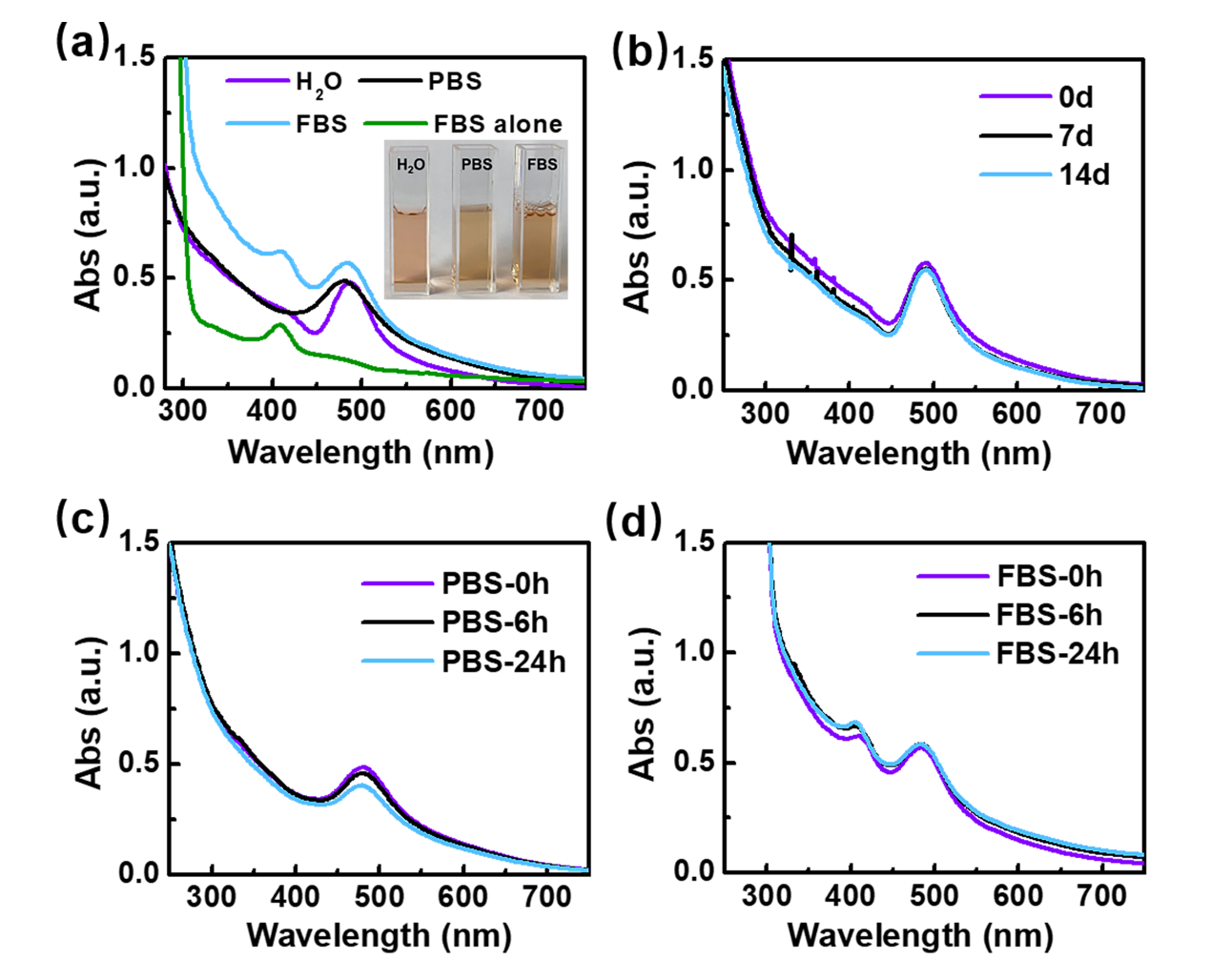


**Figure S2.** (a) The absorption spectra of the Ag_14_ clusterzymes in water, PBS and FBS solutions. Inset: photographs of Ag_14_ clusterzymes in various solutions under visible light. The stability absorption spectra of the Ag_14_ clusterzymes (b) in water, (c) PBS and (d) FBS solutions.


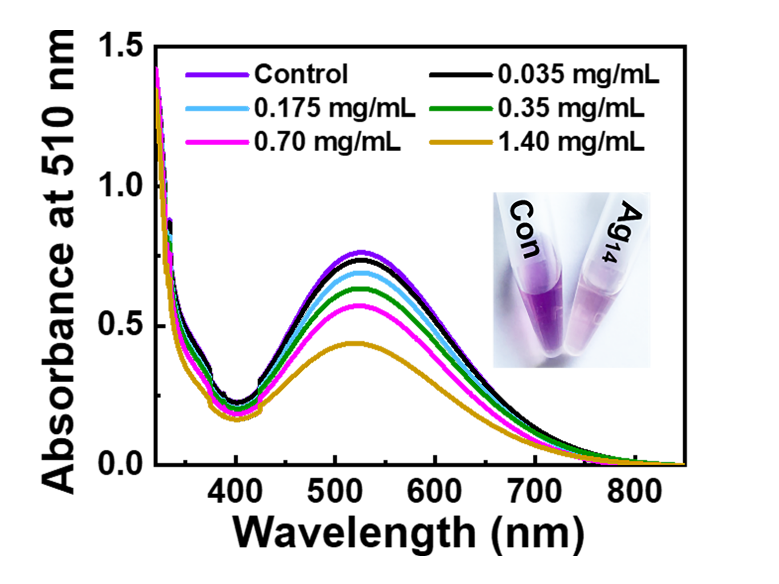


**Figure S3.** Hydroxyl radical scavenging ability of Ag_14_ clusterzymes at different concentrations characterized by SA assays.


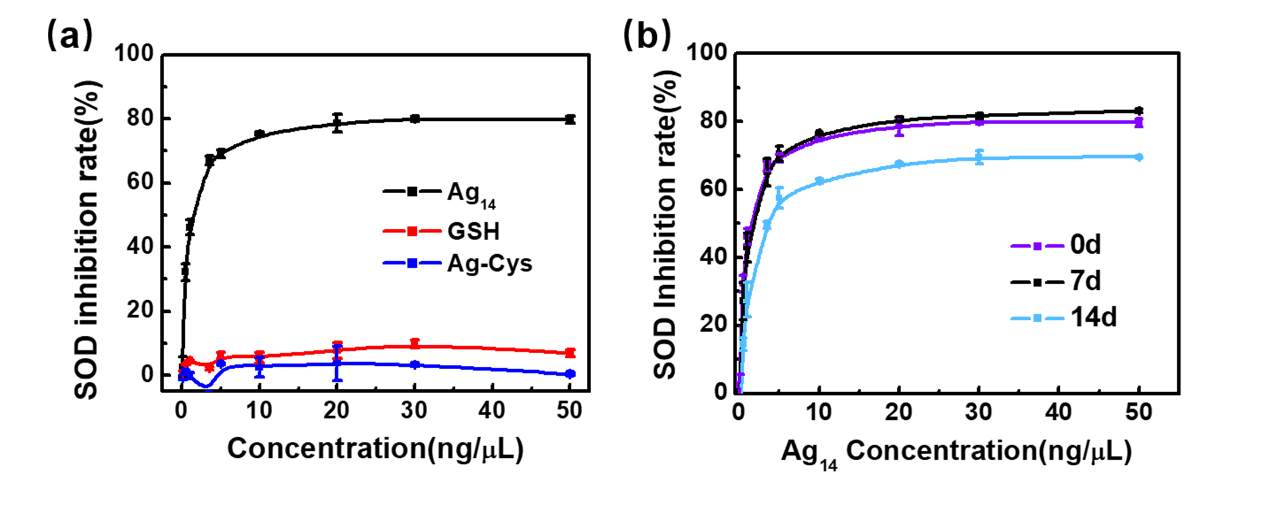


**Figure S4.** (a) The SOD-like catalytic activity of Ag_14_ clusterzymes, free GSH and Ag-Cys clusters. n=3. (b) The SOD-like catalytic stability of Ag_14_ clusterzymes as-prepared, after 7 days and 14 days. n=3. It need to be noted that the concentration of GSH is the same with Ag_14_(SG)_8_ clusterzymes.


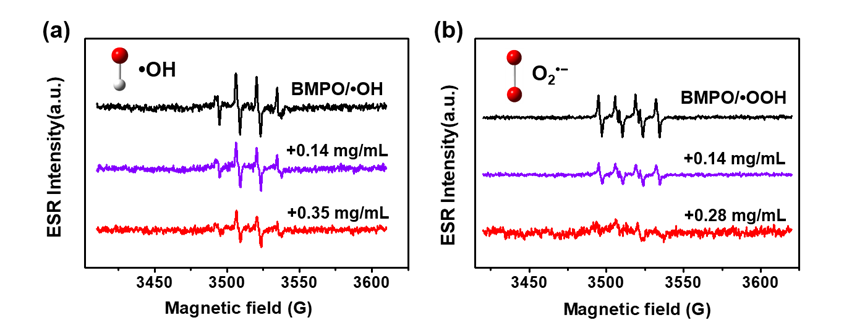


**Figure S5.** ROS scavenging activities of Ag14 clusterzymes for (a) •OH and (b) O_2_^•−^ studied by the ESR spectroscopy. BMPO is used as the spin trap for the specific trapping of •OH and O_2_^•−^ radicals.


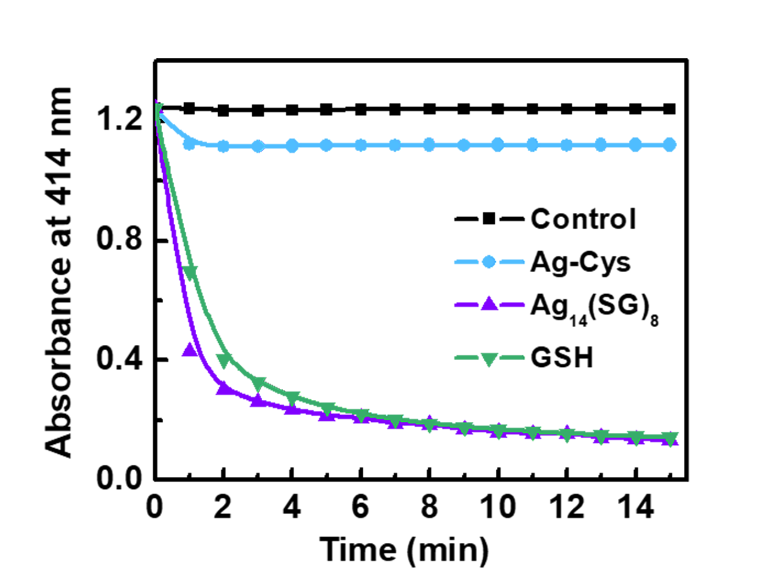


**Figure S6.** The ABTS^+•^ clearance kinetic of Ag_14_ clusterzymes, GSH and Ag-Cys nanocluter, which represent the total antioxidant capacity.


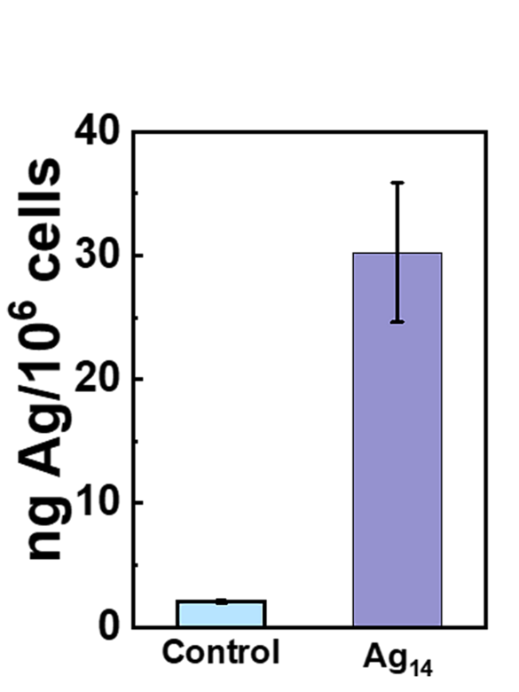


**Figure S7.** Cellular uptake of Ag_14_ clusterzymes after one hour incubation with a concentration of 280 μg/mL. The Ag concentrations were determined by ICP-MS. n = 3.


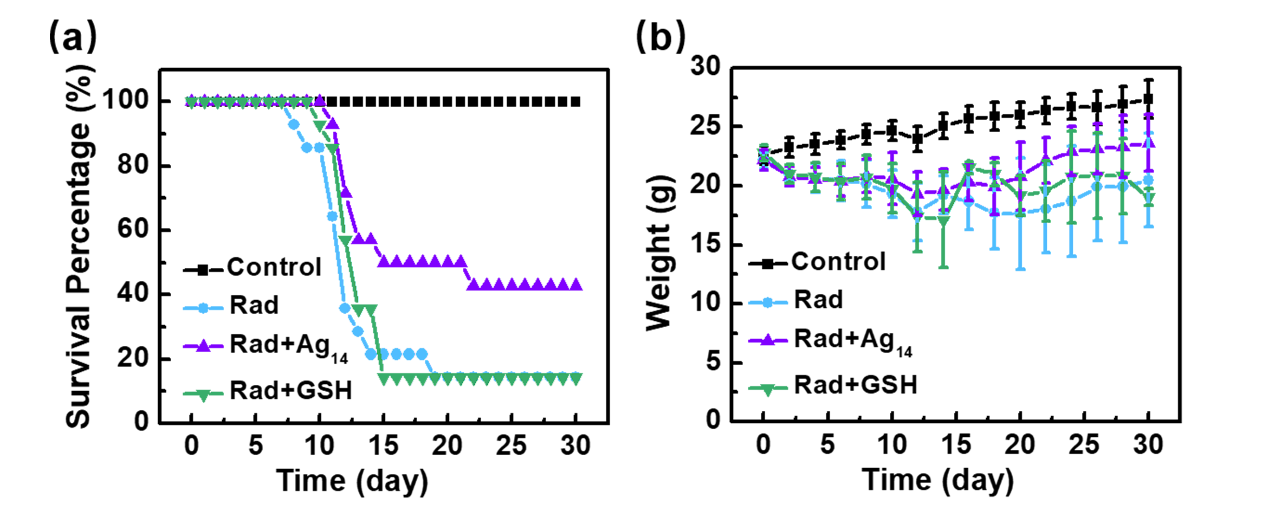


**Figure S8.** Radioprotection of GSH stabilized Ag_14_(SG)_8_ clusterzymes and free GSH *in vivo*. **a)** Survival curves of mice with or without pre-injection of Ag_14_ clusterzymes (1.4 mg/mL, 0.2 mL) or GSH (150 mg/mL (180 times the concentration of GSH in Ag_14_(SG)_8_ clusterzymes), 0.2 mL) after 7 Gy radiation (14 mice per group). **b)** Body weight of mice in different groups during 30 days.


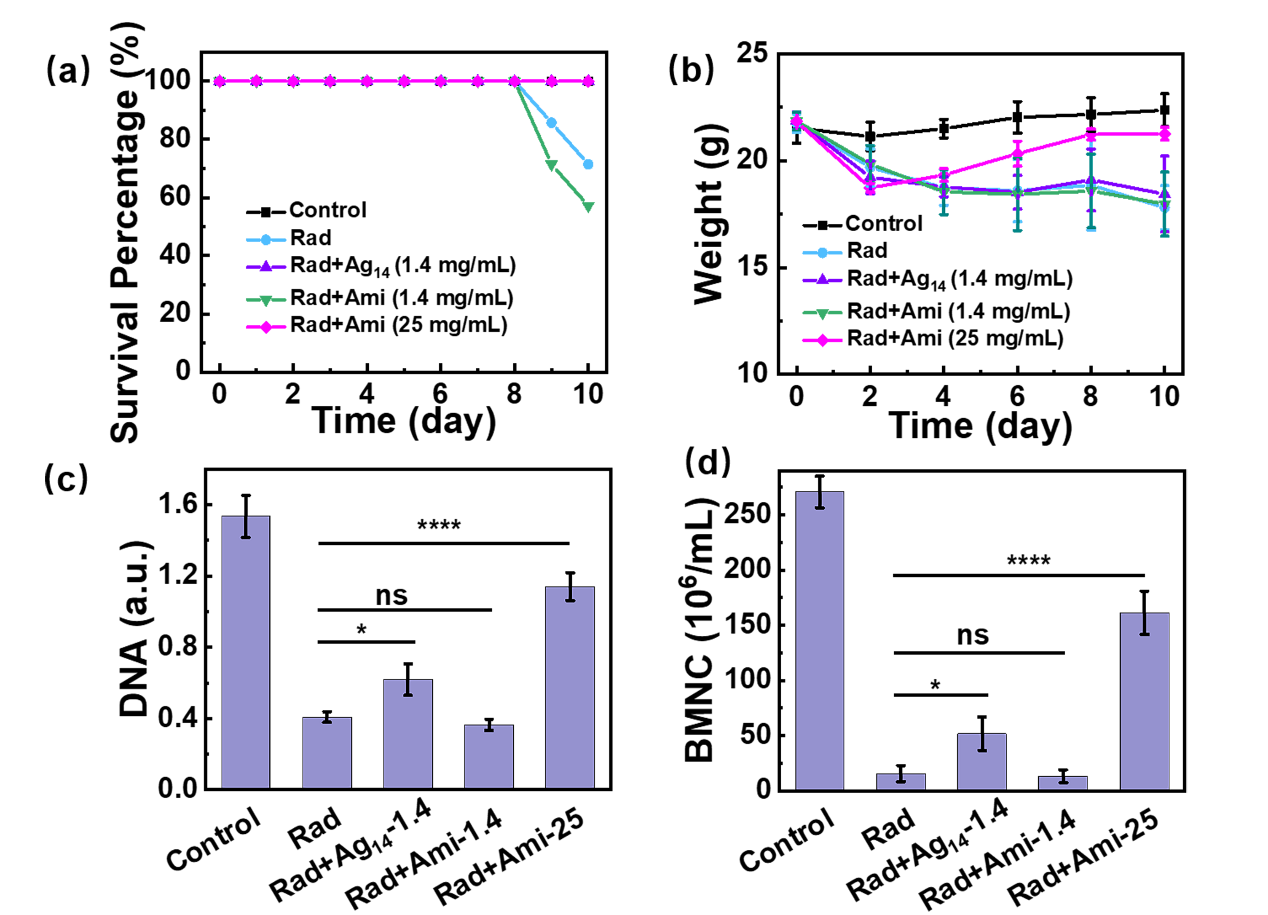


**Figure S9.** Radioprotection of Ag_14_ clusterzymes *in vivo*. a) Survival rates of healthy mice, irradiated mice, and mice irradiated pretreated with Ag_14_ clusterzymes (1.4 mg/mL, 0.2 mL), amifostine (1.4 mg/mL, 0.2 mL), and amifostine (25 mg/mL, 0.2 mL). During the observation period of 10 days, the survival rates of these 5 groups were 100%, 71%, 100%, 57% and 100% respectively (n=7). b) Body weight of mice in different groups during 10 days. c) Bone marrow total DNA content of mice in different groups at 10 days, as measured by UV−vis absorption at 268 nm. d) Counts of bone marrow nucleated cells in different groups at 10 days. P values: ***p < 0.001, **p < 0.01, or *p < 0.05, ANOVA.


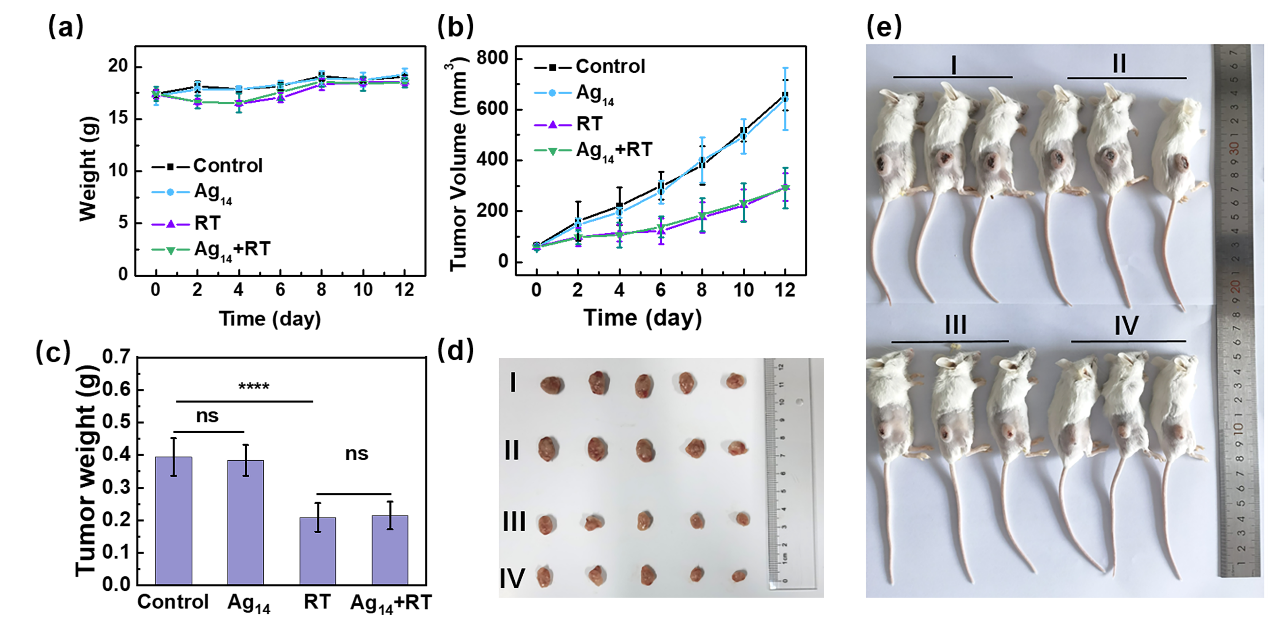


**Figure S10.** Effect on *in vivo* tumor radiotherapy. Mice were randomly divided into four groups. Ⅰ: control group; Ⅱ: Ag_14_ group; Ⅲ: RT group; Ⅳ: Ag_14_+RT group. a) Body weight of mice in different groups during 12 days. b) Tumor growth curves after radiation with pre-injection of Ag_14_ clusterzymes (5 mice per group). c) Tumor weights of different groups collected at Day 12 after various treatments. d) and e) Photograph of tumor tissues and mice bearing tumor at Day 12 after various treatments indicated. P values: ***p < 0.001, **p < 0.01, or *p < 0.05, ANOVA.


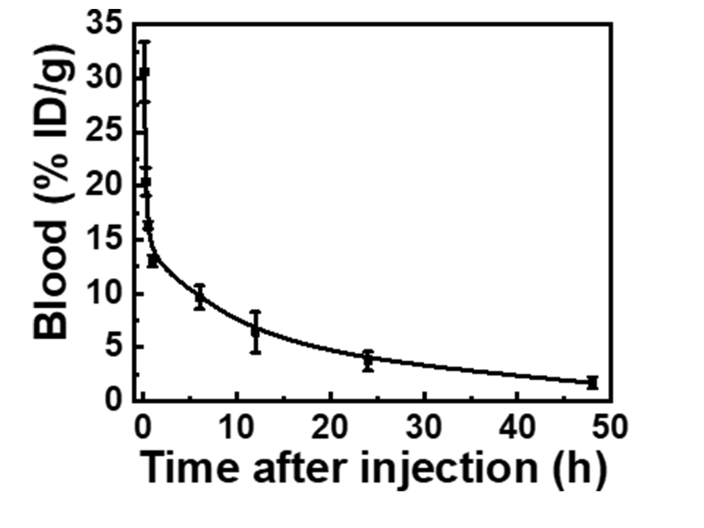


**Figure S11.** Blood concentrations of Ag_14_ clusterzymes at different time points after intravenous injection. n=3. The half-life of nanozymes in blood is about 46 min.
